# Bayesian Approach to Psychotherapy Integration: Strategic Modification of Priors

**DOI:** 10.3389/fpsyg.2019.00356

**Published:** 2019-02-26

**Authors:** Valery Krupnik

**Affiliations:** Naval Hospital Camp Pendleton, Camp Pendleton, CA, United States

**Keywords:** psychotherapy integration, nested hierarchy, bayesian brain, priors, free-energy

## Abstract

Integrative psychotherapies have become the mainstay in mental health care. The most researched therapy, CBT, being integrative itself, continues to integrate such new elements as mindfulness, spirituality, and experiential techniques. There is no commonly accepted strategy for psychotherapy integration. New elements are sometimes added on a trial and error basis with a following *post-hoc* theoretical and empirical justification. Other times, they are incorporated based on an *ad-hoc* theoretical premise, and empirical studies follow to support or invalidate it. Nevertheless, four main integrative strategies have been identified as technical eclecticism, common factors integration, principle-based assimilative integration, and theoretical integration (Norcross, 2005). Strategies outside of these four have also been suggested. Recently, a principle of nested hierarchy has been proposed as a way of integrating different strategies into a general roadmap for building an integrative therapy (Krupnik, 2018). The nested hierarchy principle does not, however, offer a strategy for theoretical integration at the top of its hierarchy. In this report, I suggest using the Bayesian theory of psychopathology for such strategy. I propose to apply Bayesian framework to psychotherapy integration and discuss a possibility of using it as a universal strategy called Strategic Modification of Priors (SMOP). I illustrate SMOP's application with a synopsis of a clinical case.

Traditionally, four strategies have been identified for psychotherapy integration: technical eclecticism, common factors integration, principle-based assimilative integration, and theoretical integration (Norcross, 2005). In short, technical eclecticism refers to construction of sets of interventions from different therapies based on the interventions' perceived or proven utility and compatibility without concern for congruence of the underlying theories. Selection of the interventions is guided by an assessment of the patient's needs, strengths, and vulnerabilities. Common factors integration is based on universal principles of change common to different therapies. Interventions are chosen to engage those principles, e.g., empathic therapeutic disposition, collaborative working alliance, motivational stance. Theoretical integration seeks to integrate different theories of psychotherapy into a unified one, which then guides the selection and design of therapeutic techniques. Although similar to theoretical integration (and not always differentiated from it), principle-based assimilation differs in that the main treatment-guiding theory assimilates as subordinate elements from other theories. There are also strategies outside the above categories.

It is unclear whether all therapies can be integrated; moreover, there is no evidence for the superiority of integrative therapies over “monotherapies.” Perhaps, the main promise of integrative treatments is in their versatility and adaptability to the needs and characteristics of the patients, which would call for comparison not as much between integrative and “mono” therapies' efficacy but between integrative and “mono” therapists' effectiveness.

## Nested Hierarchy Principle of Psychotherapy Integration

**Figure 1:**
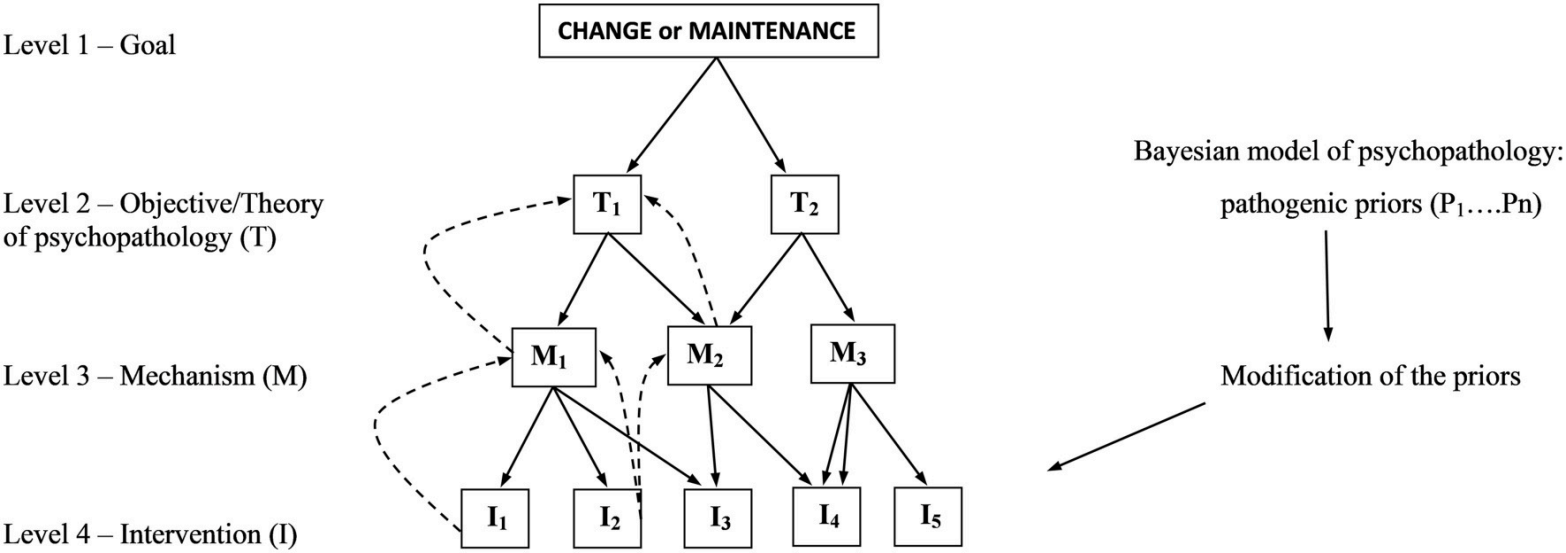
Startegic modification of priors as an organizing principle for nested hierarchy of psychotheraphy integration. Downward solid arrows indicate top-down decision/choice pathways; upward dotted arrows indicate feedback validation (modified from Krupnik, 2018).

More recently, a universal approach based on the principle of nested hierarchy (NH) has been proposed for psychotherapy integration (Krupnik, 2018). In short, an integrative therapy is constructed as a nested hierarchy of theories about the therapeutic encounter, where the higher order theories determine a set of theories at the level below (Figure 1). On top is the binary choice between change and maintenance as the goal of therapy. One level down is a theory of psychopathology that explains the patient's presentation including the symptoms and relational style (commonly referred to as case conceptualization). That theory determines both the therapeutic objectives and the lower level theories about the likely mechanism/s of change. The working theory of the mechanism/s of change then determines the choice of intervention/s at the lowest level of the hierarchy (Figure 1). The NH principle stipulates that for an effective integration the lower level theories should be consistent with the higher level ones. It also suggests that integration happens during a therapeutic encounter in the “integrate-as-you-go” way, which implies that integrative therapies may be as singular, as are therapeutic encounters.

Most, if not all, therapies may be considered integrative, because mono-technique therapy is an abstraction rather than reality. Even pharmaco-therapy includes therapeutic relationship as an active ingredient (McKay et al., 2006). It probably makes more sense to speak about the degree and limitations of integration rather than the binary choice of mono vs. integrative therapies.

High in the hierarchy, a theory of psychopathology determines the rest of the nest (Figure 1). Therefore, a unified theory of psychopathology would be an effective heuristic tool for guiding the lower level theories. The Bayesian model of psychopathology may be such a theory.

## Bayesian Model of Psychopathology

The Bayesian theory of the brain considers it a predictive coding machine [reviewed e.g., in Clark (2013)]. Contrary to the traditional stimulus-response paradigm it regards the brain as an active information-seeking and processing organ that continuously runs a generative model of the environment, both internal (the body) and external (the word). The model makes predictions about sensory stimuli by inferring their causes. Such predictions are called *priors*; they direct the top-down flow of information to guide perception and action. The bottom-up information (interoceptive from the body, exteroceptive from the world) is then compared to the predictions, and the discrepancy constitutes a prediction error. Such errors update the priors, increasing the model's accuracy. Thereby, the model directs perception and action to sample the environment to test its predictions. The model can suppress prediction errors by either adjusting the priors (through perception) or controlling the sensory input (through action), or both. This process is called active inference.

The brain implements active inference by hierarchical information processing [e.g., in Friston et al. (2014)]. For example, looking at an emoticon (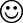) one's higher level cortical representation is that of a face symbol with a prior belief that it should contain such lower level representations as a curved line under two small circles inside a bigger circle. The inference here is that these attributes cause the perception of a “happy face” (this creates the “emoticon” category). This prior is signaled through the deep pyramidal neurons down to the primary visual cortex, where such representations are encoded. As the eyes collect sensory input, it ascends through the superficial pyramidal neurons delivering feedback to the higher level deep pyramidal neurons, comparing the input with the prediction, thus generating a prediction error. The brain then suppresses the error by either updating the prior (e.g., a straight “mouth” line would update the model to include non-emotional emoticons 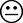 or to ascribe an emotion to it) or fulfilling it by finding or imagining/hallucinating confirming details (e.g., a slant in the straight line).

Suppressing prediction errors through recurrent cycles of active inference, the brain minimizes the uncertainty (variational free-energy), thus decreasing the entropy of its sensory states (Friston, 2010). Free-energy minimization is considered the basic mechanism/motivation for the brain (and any adaptive self-organizing system) to avoid states of uncertainty, thus maintaining homeostasis and avoiding disorder/decay. Consequently, the free-energy principle has been suggested as a unified brain theory (Friston, 2010), where anything the brain does serves to minimize its free-energy.

If the free-energy principle is a unified brain theory then malfunction of active inference may be considered a unified theory of psychopathology (Friston et al., 2014). Indeed, a growing number of psychiatric conditions have been framed in Bayesian terms, including psychosis (Powers et al., 2017), depression (Barrett et al., 2016; Badcock et al., 2017), disorders of personality (Moutoussis et al., 2014), autism (Lawson et al., 2014), functional neurological disorders (Edwards et al., 2012).

The subject of precision is at the core of the Bayesian view of psychopathology. Priors are probability density functions representing prior learning, whose inverse variance is the measure of their precision. In the emoticon example, a highly precise prior may not accommodate a circle (but only an open line) as a representation of “mouth,” whereas a less precise prior may. Likewise, a highly precise input (frequent occurrence of circular “mouths”) will have a greater impact on the prior. Therefore, precision-weighting is instrumental in determining the relative influence of top-down (prediction) and bottom-up (error) signals. Precision-weighting is mediated by neuromodulatory control of the synaptic gain of prediction error units (Friston et al., 2014). Over- or under-weighted prediction errors can reinforce false/inaccurate inference, thus leading to pathology.

People with psychotic features were shown to form hyper-precise predictions about sensory experiences, as manifested by their higher rate of cued hallucinations (Powers et al., 2017). Moreover, this effect correlated with the severity of psychosis. In other work, mood has been conceptualized as a hyperprior that weights the salience of sensory input (Clark et al., 2018). Accordingly, low precision of the mood prior in depression is thought to result in over-weighted stress-induced interoceptive input; conversely, the hyper-precise mood prior in mania results in under-weighting of such input. A more detailed account of the role of precision in functional neurological disorders is given in the “case illustration section.” As a unified theory of psychopathology, the Bayesian model is a fitting choice for the level 2 theory in the nested hierarchy of integrative therapy (Figure 1).

## Bayesian View of Psychotherapy Integration

Various psychiatric treatments have been re-conceptualized according to the Bayesian theory of psychopathology. In depression, several aspects of active inference have been identified as putative targets for established therapies (Barrett et al., 2016). One is the priors embodied by the tonic activity of the limbic cortex that depress motivation and energy. The authors suggest that deep brain stimulation of limbic connections increases the priors' precision, leading to filtering out of the “noise” stress. Another, bottom-up, way of attenuating such “noise” is by increasing the signal-to-noise ratio of the positive interoceptive input through vagal nerve stimulation (another effective treatment for depression). Cognitive behavioral therapy is thought to effect change of the higher level cognitive priors, which then guide active inference to sample the environment in new ways, thus creating new “empirical priors.”

As a unified theory of psychopathology, the Bayesian model organizes the hierarchy of integrative therapy (Figure 1) by first directing therapeutic encounter toward identification of the pathogenic priors. This can be accomplished by different means including psychodynamic analysis of the most salient defenses, which can reveal the core hyper-precise maladaptive priors. Alternatively, they can be identified in cognitive therapy as the core beliefs, or as the most rigid maladaptive behavioral patterns in behavioral analysis. Once identified, the core priors will point to mechanisms of change that could trigger their modification. Then, interventions will be chosen (or designed) to carry out such modification (Figure 1).

One way to modify a prior is by introducing a high-precision prediction error. For example, if prior A under certain circumstances triggers symptom B (a behavior, emotion, or thought), substituting B for a behavior/emotion/thought C under the same circumstances will generate a prediction error with a potential of decreasing the prior's precision and, consequently, attenuating symptom B. Such an approach has been dubbed strategic symptom displacement (SSD) and is described in detail in the next section. Another way is to manipulate the precision of the target prior or/and prediction error as in the above-described interventions for depression (Barrett et al., 2016). Thus, strategic modification of priors (SMOP) may serve as a *universal* integrative therapy guiding intervention selection (Figure 1).

Notably, SMOP may itself be viewed as active inference. In the NH/SMOP generative model of therapeutic encounter, the higher level theories of psychopathology function as priors guiding the lower level theories down to the choice of interventions, whereas the patient's response generates prediction errors that update the model. Thus, SMOP proceeds as an iterative process of active inference leading to minimization of the model's free-energy, which is expected to reinforce (increase the precision) effective and disengage ineffective interventions. This active inference perspective underscores the suggestion that psychotherapy integration is co-created in the encounter (Krupnik, 2018).

NH therapy has not yet been empirically tested, although an approach to such a test has been outlined (Krupnik, 2018); nor have SMOP and SSD been researched beyond a case study. The main hypothesized advantage of SMOP for clinicians is that unlike brand therapies it is not prescriptive but heuristic. It allows for the “integrate-as-you-go” approach (Krupnik, 2018), which is expected to help clinicians dynamically and flexibly use all their skills (thus maximizing their efficacy) in accordance with their model of a therapeutic encounter. SMOP also relaxes the mutual constraints of disparate theories on therapy integration, since interventions–not theories or therapies–are integrated. In this sense, SMOP can be regarded as “self-organizing eclecticism.”

The said advantage contains SMOP's main limitation and challenge for the therapist. A generative model of a therapeutic encounter will be as effective as it is successful in balancing the precision of its priors with that of patient-generated prediction errors. In this regard, the therapeutic skills of active listening and emotional attunement come indispensable.

## Case Illustration

An application of SMOP and SSD has recently been reported in a case of motor functional neurological disorder (Krupnik and Cherkasova, 2018). The Bayesian theory of functional neurological symptoms, both sensory and motor, regards them as perceptions or movements “fulfilling” the corresponding priors (Edwards et al., 2012). This happens when a prior about the cause of an abnormal sensation or movement (or lack thereof) becomes so rigid (hyper-precise) that prediction errors fail to correct it. For example, weakness in a limb may result in a prediction in the somatosensory cortex of inability to perform the volitional movement. Such failure may then be consciously interpreted as a symptom, and once that happens, a functional paralysis may develop to “fulfill” the prior. According to this theory, abnormal priors are formed at several levels from conscious interpretation of the symptom through the motor cortex that fails to initiate the movement in order to fulfill the higher order prior. Such malfunction of active inference is in the service of minimizing the free-energy of the corresponding neural circuitry because as long as the priors are confirmed, the level of uncertainty (and free-energy) is kept low. This may explain the high resistance of functional symptoms to treatment and the lack of commonly accepted effective therapies.

The case in question involves involuntary shakes of the head and shoulders that sometimes would generalize to whole-body convulsions. A thorough assessment failed to identify any neurological abnormalities, and the symptoms were concluded to be functional. After trials of multiple therapies including, among others, cognitive behavioral therapy, relaxation and mindfulness training, and pharmacotherapy failed to resolve or attenuate the symptoms for 2 years, a SMOP approach was implemented. The therapist first identified priors that were plausible contributors to the symptoms. From history taking, it appeared that the shakes started as a “ducking” motion during exposure to danger (explosions). Later, in absence of the danger, this motion became associated with stress and self-awareness and was accounted for by the patient as an involuntary symptom, a “tic” (of note, he did not meet the criteria for a tic disorder). The therapist conceptualized this motor symptom as “fulfilling” the somatosensory expectation of the “tic” in response to stress (in the same way an ordinary stress response includes increased heart rate and muscle tension). At the conscious level, the prior was identified as an expectation of the movements to be involuntary to fulfill their role as a symptom outside the patient's control.

A two-prong (top-down and bottom-up) strategy was used to target the identified priors by introducing and amplifying prediction errors at both the cognitive and behavioral levels. The therapist made use of an observation (common for functional symptoms) that greater attention would trigger and amplify the symptoms, while in its absence they remained inactive. This observation helped decrease the precision/certainty of the belief in the “tic”s' uncontrollability and develop a modified belief in its psychological nature as part of the patient's stress response.

A breakthrough in treatment had not, however, come until a bottom-up behavioral intervention was implemented. In order to introduce a prediction error at the behavioral level, the patient was encouraged to regularly practice a substitute behavior in the form of repetitive tying-releasing rope knots when under stress. Within a month after the start of such practice, the head and shoulder shakes decreased precipitously, and the whole body convulsions stopped.

SSD appears similar to habit reversal training (Azrin and Nunn, 1973). There is, however, an important difference. Unlike habit reversal training, SSD *does not* require substitute behavior be incompatible with the symptom, which may have the advantage of avoiding resistance. I want to emphasize that the target of SSD is not as much the symptomatic behavior as the prior it fulfills.

Understood broadly, a symptom may manifest as behavior, emotion, and thought. Therefore, SSD can be applied at any level, as long as the respective priors are properly identified. Its effectiveness will largely depend on how precisely the pathogenic priors are identified and how effectively the substitute action introduces prediction error.

## Conclusion

In this report, I propose NH/SMOP as a universal heuristic (as opposed to prescriptive) strategy for constructing integrative psychotherapy. This strategy is based on the Bayesian view of both psychopathology and therapeutic encounter and proposes an integrative therapy be co-created in an active inference process. SMOP is expected to allow for application/design of interventions that is highly versatile and responsive to the needs of a therapeutic encounter.

## Author Contributions

The author confirms being the sole contributor of this work and has approved it for publication.

### Conflict of Interest Statement

The author declares that the research was conducted in the absence of any commercial or financial relationships that could be construed as a potential conflict of interest.
